# Clinical Utility of the Triglyceride–Glucose Index in Assessing Hepatic Steatosis Severity Within the MASLD Spectrum

**DOI:** 10.3390/diagnostics16060872

**Published:** 2026-03-15

**Authors:** Ömer Faruk Alakuş, İhsan Solmaz, Jehat Kiliç, Sedat Çiçek

**Affiliations:** 1 Department of Internal Medicine, Gazi Yaşargil Training and Research Hospital, University of Health Sciences, Diyarbakır 21070, Türkiye; ihsan2157@gmail.com; 2 Rheumatology, Department of Internal Medicine, School of Medicine, Fırat University, Elazığ 23119, Türkiye; jehat_kilic@outlook.com; 3 Gastroenterology, Department of Internal Medicine, School of Medicine, Fırat University, Elazığ 23119, Türkiye; drsedatcicek23@gmail.com

**Keywords:** triglyceride–glucose index, hepatic steatosis, inflammation, metabolic dysfunction-associated steatotic liver disease

## Abstract

**Background/Objectives**: The global increase in metabolic dysfunction-associated steatotic liver disease underscores the need for accessible and reliable markers to assess hepatic steatosis. The triglyceride–glucose (TyG) index, derived from fasting plasma glucose and triglyceride levels, has emerged as a practical surrogate marker of insulin resistance and has been increasingly associated with metabolic liver involvement. This study aimed to evaluate the relationship between the TyG index and the severity of hepatic steatosis assessed by ultrasonography. **Methods**: This retrospective cross-sectional study included 480 adult patients without a prior diagnosis of diabetes mellitus or hypertension who underwent fasting laboratory testing and abdominal ultrasonography between January 2024 and May 2025. Fasting plasma glucose and triglyceride levels were obtained on the same day as ultrasonographic evaluation. Hepatic steatosis was assessed by a single experienced radiologist using standardized ultrasonographic criteria, and patients were categorized into three groups according to steatosis grade (grade 0, grade 1, and grade 2–3; *n* = 160 for each group). Demographic data and laboratory parameters, including glucose, triglycerides, HbA1c, platelet count, neutrophils, lymphocytes, monocytes, ALT, AST, and total cholesterol levels, were recorded. The TyG index was calculated using the formula: TyG = ln[(fasting triglycerides × fasting glucose)/2]. **Results**: A total of 480 patients (30.6% male) were included in the analysis. Mean fasting glucose, triglyceride, and TyG index values were 94.20 ± 11.15 mg/dL, 146.91 ± 83.94 mg/dL, and 8.70 ± 0.55, respectively. Metabolic and inflammatory parameters increased significantly with advancing steatosis grades (all *p* < 0.05). The TyG index demonstrated a clear stepwise increase from grade 0 (8.29 ± 0.42) to grade 1 (8.74 ± 0.42) and grade 2–3 steatosis (9.07 ± 0.49) (*p* < 0.001), with all pairwise comparisons remaining statistically significant. Receiver operating characteristic (ROC) analysis showed good discriminative performance of the TyG index for hepatic steatosis (AUC = 0.829), and an optimal cutoff value of 7.90 was identified using the Youden index, yielding high sensitivity for detection. In multivariable logistic regression analysis, the TyG index remained the strongest independent predictor of hepatic steatosis (adjusted OR 11.41, 95% CI 6.10–21.34; *p* < 0.001). **Conclusions**: The TyG index increased progressively with the severity of hepatic steatosis and showed strong associations with metabolic and inflammatory parameters. These findings support the TyG index as a simple and accessible marker reflecting metabolic dysfunction and hepatic steatosis, with potential value for early risk stratification in clinical practice.

## 1. Introduction

Metabolic dysfunction-associated steatotic liver disease (MASLD) has recently been established as the definitive term encompassing liver disorders associated with metabolic dysfunction, replacing the previous nomenclature of non-alcoholic fatty liver disease (NAFLD) [1]. It is estimated that more than 30% of the global adult population is affected by MASLD, and its prevalence continues to rise alongside increasing rates of obesity, type 2 diabetes mellitus (T2DM), and metabolic syndrome [1,2].

MASLD is defined by the presence of hepatic steatosis together with at least one cardiometabolic risk factor. According to current consensus criteria, these cardiometabolic abnormalities include overweight or obesity (body mass index [BMI] ≥ 25 kg/m^2^, or ≥23 kg/m^2^ in individuals of Asian ethnicity) or increased waist circumference (≥94 cm in men and ≥80 cm in women in European populations), dysglycemia or type 2 diabetes mellitus (prediabetes defined as HbA1c 5.7–6.4% or fasting plasma glucose 100–125 mg/dL), dyslipidemia (plasma triglycerides ≥ 150 mg/dL or reduced high-density lipoprotein cholesterol < 40 mg/dL in men and <50 mg/dL in women), and elevated blood pressure (≥130/85 mmHg) or the use of antihypertensive treatment [1]. These cardiometabolic abnormalities reflect the central role of insulin resistance and metabolic dysfunction in the pathogenesis of MASLD. The disease may progress along a spectrum ranging from simple steatosis to metabolic dysfunction-associated steatohepatitis (MASH), fibrosis, cirrhosis, and ultimately hepatocellular carcinoma (HCC) [3]. Because MASLD is frequently asymptomatic in its early stages, early identification of individuals at risk and appropriate metabolic management are essential to prevent long-term hepatic and cardiovascular complications [4].

Among the metabolic abnormalities involved in MASLD, insulin resistance is considered the key pathophysiological mechanism driving both disease development and progression [5]. By increasing adipose tissue lipolysis and hepatic free fatty acid influx, insulin resistance promotes de novo lipogenesis and triglyceride accumulation within hepatocytes [6]. As the severity of hepatic steatosis increases, it often mirrors the magnitude of underlying metabolic dysfunction [5]. Although liver biopsy remains the historical gold standard for diagnosing and staging MASLD, its invasiveness, cost, and risk of complications substantially limit its utility for routine assessment and large-scale screening [5].

In daily clinical practice, abdominal ultrasonography (USG) is the most widely utilized first-line imaging modality for the evaluation of hepatic steatosis due to its accessibility and low cost [1]. Ultrasonography allows grading of steatosis severity based on hepatic echogenicity, visualization of the diaphragm, and the clarity of intrahepatic vascular structures. However, this technique is inherently operator-dependent, and its sensitivity decreases in patients with mild steatosis or elevated BMI [5]. These limitations underscore the need for objective, non-invasive, and cost-effective biomarkers capable of reflecting steatosis severity and complementing imaging-based assessment.

The TyG index, calculated from fasting triglyceride and fasting plasma glucose levels, has gained recognition as a reliable surrogate marker of insulin resistance [6]. Beyond hepatic steatosis, accumulating evidence indicates that the TyG index is associated with a wide range of metabolic and systemic diseases, including diabetes, cardiovascular disorders, cerebrovascular diseases, and kidney disease [7]. Unlike the Homeostasis Model Assessment for Insulin Resistance (HOMA-IR), the TyG index does not require insulin measurements, enhancing its feasibility for routine clinical practice. Recent studies have demonstrated that the TyG index and its anthropometric derivatives, such as TyG-BMI and TyG-waist circumference, exhibit high diagnostic accuracy for identifying hepatic steatosis within the MASLD spectrum [6,8,9]. However, although several studies have demonstrated associations between the TyG index and related composite indices (such as TyG-BMI and TyG–waist circumference) and the presence of hepatic steatosis, data evaluating their relationship with the severity of hepatic steatosis across different steatosis grades remain limited.

The present study aimed to evaluate the clinical utility of the TyG index in assessing hepatic steatosis severity within the MASLD spectrum. Specifically, we investigated whether TyG index values increase progressively across ultrasonographic steatosis grades and assessed the potential of the TyG index as an independent marker of steatosis severity. By clarifying this relationship, we aimed to determine whether the TyG index could serve as a simple, non-invasive biomarker reflecting steatosis severity and supporting metabolic risk stratification in clinical practice.

## 2. Materials and Methods

The study protocol was approved by the University of Health Sciences Diyarbakır Gazi Yaşargil Training and Research Hospital Clinical Research Ethics Committee on 13 June 2025 (approval number: 508). Due to the retrospective nature of the study, the requirement for informed consent was waived. All procedures were conducted in accordance with the ethical principles of the Declaration of Helsinki.

**Figure 1:**
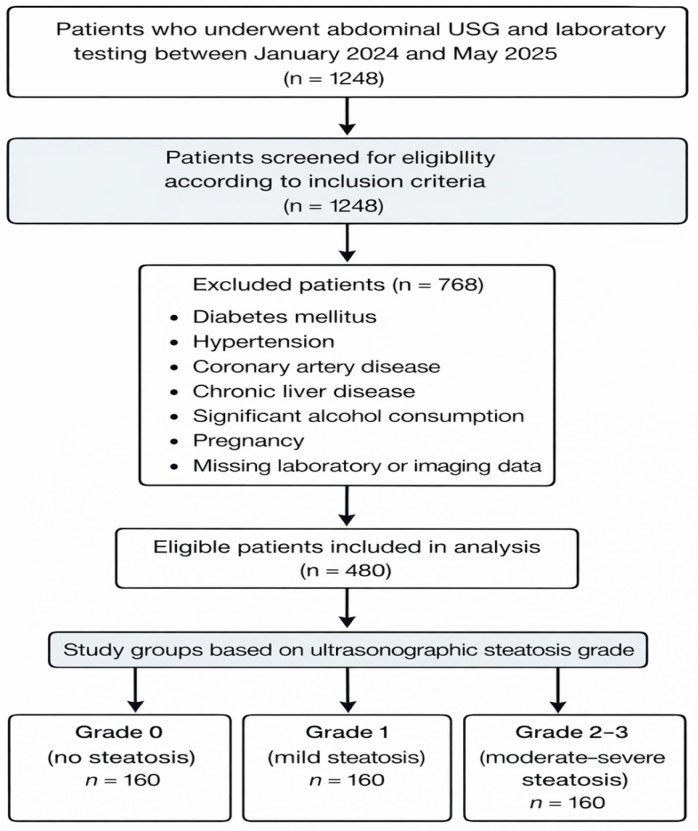
Flow diagram of patient selection and study group formation.

This retrospective cross-sectional study included a total of 480 adult patients evaluated at the Department of Internal Medicine, University of Health Sciences, Diyarbakır Gazi Yaşargil Training and Research Hospital, Diyarbakır, Turkey. Demographic data, laboratory findings, and imaging records were retrieved retrospectively from the hospital’s electronic medical record system. Patients who underwent laboratory testing and abdominal ultrasonography between January 2024 and May 2025 were screened for eligibility. The study population consisted of patients evaluated in the internal medicine outpatient clinics of our hospital. Hepatic steatosis was graded using standardized ultrasonographic criteria as described in previous studies. The overall patient selection process is illustrated in the study flow diagram (Figure 1).

Flow diagram illustrating the patient selection process. A total of 1248 patients who underwent abdominal ultrasonography and laboratory testing between January 2024 and May 2025 were screened. After applying the exclusion criteria, 480 eligible patients were included in the final analysis and categorized according to ultrasonographic steatosis grade.

### 2.1. Study Population and Data Collection

Patients aged 18 years and older were eligible for inclusion. Individuals with a prior diagnosis of diabetes mellitus or hypertension were excluded in order to minimize the potential confounding effects of advanced metabolic disease on TyG index levels and hepatic steatosis severity. Additional exclusion criteria included a history of coronary artery disease or established chronic liver diseases (including viral hepatitis, autoimmune or cholestatic liver diseases), significant alcohol consumption (defined as ≥30 g/day for men and ≥20 g/day for women), pregnancy, and incomplete laboratory or imaging data.

Only patients who had fasting plasma glucose and triglyceride measurements obtained after at least 8 h of overnight fasting on the same day as abdominal ultrasonography were included. To minimize interobserver variability, only ultrasonographic examinations performed by a single experienced radiologist were considered eligible for analysis. Patients with ultrasonographic evidence of hepatic steatosis (grade 1 and grade 2–3) and a body mass index >25 kg/m^2^ were considered to fall within the MASLD spectrum, as overweight or obesity represents one of the recognized cardiometabolic risk factors within the current MASLD diagnostic framework, whereas individuals without ultrasonographic steatosis (grade 0) served as the control group.

Demographic data, including sex, were recorded from the electronic medical record system. Fasting plasma glucose and triglyceride levels were used to calculate the TyG index. In addition, laboratory parameters obtained concurrently with fasting plasma glucose and triglyceride measurements as part of the same clinical evaluation—including glycated hemoglobin (HbA1c), platelet count, neutrophil count, lymphocyte count, monocyte count, alanine aminotransferase (ALT), aspartate aminotransferase (AST), and total cholesterol levels—were collected to characterize the metabolic and inflammatory profiles of the study population. All biochemical parameters, including fasting plasma glucose, triglycerides, HbA1c, ALT, and AST, were measured using an automated biochemical analyzer (Abbott Architect c16000, Abbott Diagnostics, Abbott Park, IL, USA) in the central laboratory of our hospital. Complete blood count analyses, including neutrophil, lymphocyte, monocyte, and platelet counts, were performed using an automated hematology analyzer (Sysmex XN-1000, Sysmex Corporation, Kobe, Japan).

### 2.2. Ultrasonographic Assessment

Abdominal ultrasonography was performed using a Philips ultrasound system (Philips Healthcare, Amsterdam, The Netherlands) equipped with a 3.5–5 MHz convex transducer. All examinations were conducted by the same experienced radiologist, who was blinded to the laboratory results at the time of imaging evaluation.

Hepatic steatosis was assessed based on standardized ultrasonographic criteria, including increased liver echogenicity relative to the renal cortex, blurring of intrahepatic vascular margins, and posterior beam attenuation. Steatosis severity was classified as grade 0 (absence of steatosis), grade 1 (mild steatosis), and grade 2–3 (moderate to severe steatosis). For subsequent analyses, patients were categorized into three groups according to hepatic steatosis grade: grade 0 (*n* = 160), grade 1 (*n* = 160), and grade 2–3 (*n* = 160). To allow balanced statistical comparisons between groups, an equal number of patients was included in each steatosis category.

### 2.3. Triglyceride–Glucose Index

The TyG index was calculated as a surrogate marker of insulin resistance using fasting plasma glucose and triglyceride values obtained on the same day as ultrasonographic assessment. The TyG index was computed according to the widely accepted formula:

TyG index = ln[(fasting triglycerides (mg/dL) × fasting plasma glucose (mg/dL))/2]

### 2.4. Statistical Analysis

The normality of the variables was assessed using the Kolmogorov–Smirnov test. Variables that followed a normal distribution were presented as mean ± standard deviation (SD), whereas non-normally distributed variables were expressed as median (minimum–maximum). For comparisons among groups, one-way analysis of variance (ANOVA) was applied to normally distributed continuous variables, while Kruskal–Wallis tests were used for non-normally distributed variables. When global tests indicated statistical significance, Bonferroni-adjusted post hoc analyses were performed to determine pairwise group differences. Receiver operating characteristic (ROC) curve analysis was performed to assess the discriminative ability of the TyG index for the presence of hepatic steatosis. The area under the curve (AUC) and corresponding 95% confidence intervals were calculated. The optimal cutoff value was determined using the maximum Youden index. In addition, univariate and multivariable logistic regression analyses were performed to identify factors independently associated with hepatic steatosis. Multicollinearity among the independent variables was assessed using variance inflation factor (VIF) analysis. A VIF value > 5 was considered indicative of significant multicollinearity. A *p*-value of <0.05 was considered statistically significant. All statistical analyses were conducted using SPSS Statistics Version 26.0 (IBM Corp., Armonk, NY, USA).

## 3. Results

A total of 480 participants were included in the analysis, comprising 147 males (30.6%) and 333 females (69.4%). The mean fasting plasma glucose level was 94.20 ± 11.15 mg/dL (range: 70–125), while triglyceride levels showed greater variability, with a mean value of 146.91 ± 83.94 mg/dL (range: 36–390). The TyG index ranged from 7.27 to 10.89, with an overall mean of 8.70 ± 0.55. Mean HbA1c was 5.74 ± 2.33%, ranging from 4.80 to 6.50.

Hematological parameters demonstrated mean platelet, neutrophil, lymphocyte, and monocyte counts of 291.19 ± 66.88 × 10^3^/µL (range: 100–544), 4.63 ± 1.42 × 10^3^/µL (range: 1.91–9.85), 2.50 ± 0.76 × 10^3^/µL (range: 0.42–5.87), and 0.48 ± 0.15 × 10^3^/µL (range: 0.18–1.08), respectively. Regarding liver enzymes, mean AST and ALT levels were 23.84 ± 12.18 U/L (range: 8–125) and 29.26 ± 25.28 U/L (range: 4–191), respectively. The mean total cholesterol level was 192.67 ± 38.53 mg/dL, with values ranging from 54 to 331 mg/dL. Baseline demographic and laboratory characteristics of the study population are summarized in Table 1).

Across increasing steatosis grades (grade 0, grade 1, and grade 2–3), multiple metabolic and inflammatory parameters exhibited significant upward trends (Table 2). Fasting glucose levels increased progressively with steatosis severity, rising from 90.7 ± 9.0 mg/dL in grade 0 to 92 (71–122) mg/dL in grade 1 and 97 (76–125) mg/dL in grade 2–3 (*p* < 0.001). Similarly, triglyceride levels showed a marked stepwise increase across grades (86 [36–257] vs. 135 [48–373] vs. 189.5 [48–390] mg/dL; *p* < 0.001), which was paralleled by a corresponding rise in the TyG index (8.29 ± 0.42, 8.74 ± 0.42, and 9.07 ± 0.49, respectively; *p* < 0.001) (Table 2).

Glycemic status, as reflected by HbA1c levels, was significantly higher in patients with steatosis, demonstrating a gradual increase from grade 0 to grade 2–3 (5.40 [4.9–6.2] vs. 5.60 [4.9–6.50] vs. 5.76 ± 0.36; *p* = 0.002). Platelet counts also showed a modest but statistically significant increase with advancing steatosis severity (276.5 [147–477], 282 [100–544], and 288.5 [176–501] × 10^3^/µL for grades 0, 1, and 2–3, respectively; *p* = 0.038) (Table 2).

Inflammatory cell counts increased consistently across steatosis grades. Neutrophil counts rose from 4.25 [1.91–7.40] × 10^3^/µL in grade 0 to 4.51 [2.12–8.64] × 10^3^/µL in grade 1 and 4.67 [2.56–9.85] × 10^3^/µL in grade 2–3 (*p* = 0.015). Lymphocyte and monocyte counts demonstrated similar stepwise increases, with lymphocyte counts of 2.26 ± 0.65, 2.36 [0.91–4.88], and 2.81 ± 0.82 × 10^3^/µL (*p* < 0.001), and monocyte counts of 0.435 [0.20–0.99], 0.450 [0.18–1.07], and 0.480 [0.22–1.08] × 10^3^/µL across grades 0, 1, and 2–3, respectively (*p* = 0.004) (Table 2).

Liver enzyme levels demonstrated a clear stepwise elevation with increasing steatosis severity. AST levels increased from 20 [8–62] U/L in grade 0 to 21 [9–48] U/L in grade 1 and 22 [10–125] U/L in grade 2–3 (*p* < 0.001), while ALT levels rose from 17 [7–56] U/L to 21 [4–80] U/L and 28 [4–191] U/L across the respective grades (*p* < 0.001). Total cholesterol levels were also significantly higher in grade 1 and grade 2–3 compared to grade 0 (181.5 [54–331] vs. 197.9 ± 38.8 vs. 196.0 ± 34.8 mg/dL; *p* = 0.021) (Table 2).

The TyG index demonstrated a strong and graded association with steatosis severity (Table 3). Mean TyG values increased progressively from grade 0 (8.29 ± 0.42; 95% CI: 8.23–8.36) to grade 1 (8.74 ± 0.42; 95% CI: 8.68–8.81) and grade 2–3 (9.07 ± 0.49; 95% CI: 8.99–9.14). One-way ANOVA revealed a highly significant overall difference in TyG index values across the three groups (*p* < 0.001), and Bonferroni-corrected post hoc analyses confirmed that all pairwise comparisons were statistically significant, indicating a consistent increase in TyG levels with advancing steatosis severity (Table 4).

**Figure 2:**
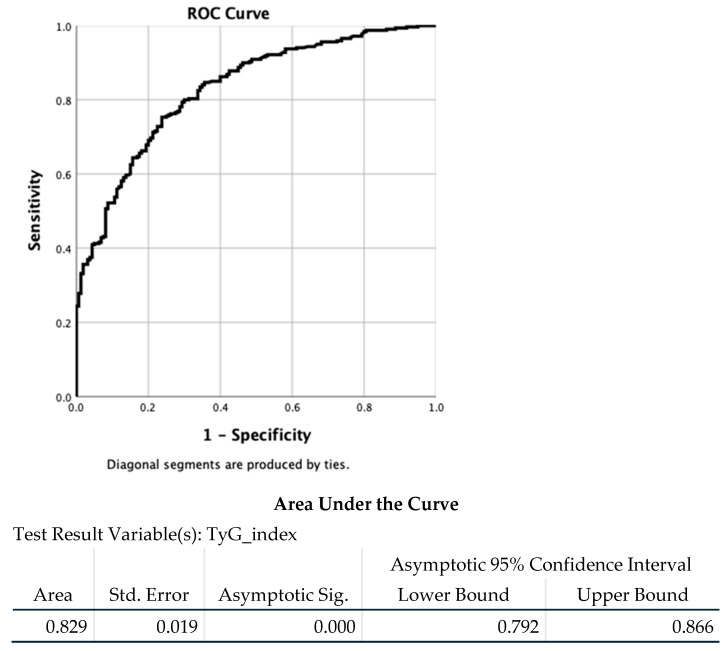
Receiver operating characteristic (ROC) curve of the TyG index for the detection of hepatic steatosis.

ROC curve analysis demonstrated good discriminative performance of the TyG index for hepatic steatosis, with an area AUC of 0.829 (95% CI: 0.792–0.866; *p* < 0.001). The optimal cutoff value, determined using the maximum Youden index, was 7.90. At this threshold, the TyG index achieved a high sensitivity of 0.984 and a specificity of 0.194, corresponding to the highest Youden index value (0.178) in the study population (Table 5, Figure 2).

The AUC was 0.829 (95% confidence interval: 0.792–0.866; *p* < 0.001), indicating good discriminative performance of the TyG index.

Univariate logistic regression analysis identified several variables associated with hepatic steatosis, including age, male sex, neutrophil count, lymphocyte count, monocyte count, HbA1c, AST, ALT, and the TyG index, whereas platelet count was not significantly associated (Table 6).

In the multivariable logistic regression model, the TyG index remained the strongest independent predictor of hepatic steatosis (adjusted OR 11.41, 95% CI 6.10–21.34; *p* < 0.001). HbA1c (adjusted OR 3.50, 95% CI 1.57–7.77; *p* = 0.002) and ALT levels (adjusted OR 1.05, 95% CI 1.02–1.07; *p* = 0.001) were also independently associated with steatosis, while the other variables were not significant after adjustment (Table 6).

## 4. Discussion

In this study, we demonstrated that the TyG index increases progressively and significantly in parallel with the ultrasonographic severity of hepatic steatosis within the MASLD spectrum. This finding is clinically relevant, as it indicates that the TyG index reflects not only the presence of hepatic steatosis but also the degree of intrahepatic fat accumulation and associated metabolic impairment. By relying on routinely available laboratory parameters, our results support the potential role of the TyG index as a practical, non-invasive marker for assessing steatosis severity and metabolic burden, without the need for invasive diagnostic procedures. Importantly, multivariable logistic regression analysis demonstrated that the TyG index remained the strongest independent predictor of hepatic steatosis even after adjustment for demographic and laboratory variables, further supporting the robustness of the observed association.

This graded association can be explained by the central role of insulin resistance in the pathophysiology of MASLD. Insulin resistance promotes enhanced lipolysis in adipose tissue, leading to increased hepatic influx of free fatty acids, which in turn stimulates de novo lipogenesis and triglyceride accumulation within hepatocytes. In line with this mechanism, we observed a clear stepwise increase in TyG index values from 8.29 in grade 0 steatosis to 9.07 in grade 2–3 steatosis, reflecting the escalating degree of metabolic dysfunction across disease severity. Moreover, the parallel elevations in liver enzymes (ALT and AST) and inflammatory cell counts (neutrophils, monocytes, and lymphocytes) with advancing steatosis grades suggest that the TyG index captures a broader pathophysiological milieu characterized by lipotoxicity and low-grade systemic inflammation accompanying MASLD progression.

Consistent with previous studies, our ROC analysis supports the diagnostic significance of the TyG index across different populations. A systematic meta-analysis conducted by Wang et al. reported an AUC of 0.75 for the TyG index in identifying MASLD, whereas our study demonstrated a higher discriminative performance with an AUC of 0.829. This value is comparable to the AUC of 0.83 reported by Yang et al. in a large Chinese population, further supporting the diagnostic relevance of the TyG index across diverse cohorts [10,11].

However, the predictive performance of the TyG index has been reported to be lower in certain specific populations. In a study focusing on Egyptian adults with type 2 diabetes, Megalaa et al. reported a substantially lower AUC of 0.637 for the TyG index alone and suggested that composite indices such as TyG-BMI, which demonstrated an AUC of 0.775, may provide improved diagnostic performance in diabetic populations [12]. In addition, Boushehri et al. reported that TyG-based indices exhibit particularly high sensitivity in female populations. This observation is consistent with the predominance of women in our cohort (69.4%) and the very high sensitivity observed at the identified threshold value (0.984) [13].

Although the TyG index demonstrated good discriminative performance for identifying hepatic steatosis, the optimal cutoff identified in this study showed very high sensitivity but relatively low specificity. This suggests that while the proposed threshold is effective in identifying most individuals with hepatic steatosis, it may also classify a substantial proportion of individuals without steatosis as positive. Therefore, this cutoff value may be more appropriate for screening purposes rather than as a definitive diagnostic threshold. In particular, the TyG index may serve as a simple and inexpensive preliminary screening tool in routine clinical practice, especially in settings where access to abdominal ultrasonography is limited or delayed, helping clinicians identify individuals who may benefit from further imaging-based evaluation.

In our multivariable logistic regression analysis, the TyG index emerged as the strongest independent predictor of hepatic steatosis, with an adjusted odds ratio of 11.41 (95% CI: 6.10–21.34). This strong independent association underscores the close relationship between insulin resistance–related metabolic disturbances and hepatic fat accumulation within the MASLD spectrum. Moreover, the magnitude of this association appears greater than that reported in several recent studies. For instance, Wu et al. reported that while the TyG index was significantly correlated with MASLD severity, it yielded lower adjusted OR values, ranging from 3.386 for mild to 5.701 for severe cases [14].

However, variability in optimal TyG cutoff values has been reported across populations. While Guo et al. proposed a higher cutoff value of 8.7 in Chinese adults, our ROC-based analysis identified a lower threshold of 7.90 [15]. This difference may be attributed to variations in study design, population characteristics, and outcome definitions. Notably, our focus on steatosis severity and early risk identification, rather than binary disease classification, suggests that lower TyG thresholds may be more appropriate for capturing metabolic risk in asymptomatic individuals before advanced hepatic or metabolic deterioration occurs.

Our observation that certain inflammatory parameters—particularly neutrophil, lymphocyte, and monocyte counts—increase with advancing steatosis severity provides further insight into the systemic inflammatory component of MASLD. Yang et al. demonstrated that the monocyte-to-lymphocyte ratio (MLR) is independently associated with MASLD risk and steatosis severity assessed by controlled attenuation parameter, while showing no significant association with fibrosis progression [16]. In a similar vein, Eid et al. reported a dissociation between circulating pro-inflammatory cytokines, such as interleukin-6 and tumor necrosis factor-α, and fibrosis severity in early MASLD, indicating that inflammatory markers may primarily reflect metabolic stress rather than structural liver damage [17].

Recent mechanistic data help contextualize these observations. Shrestha et al. highlighted the role of neutrophils in the early stages of MASLD, demonstrating that metabolic and lipotoxic stress can activate innate immune responses before the development of advanced histopathological changes [18]. These findings suggest that increases in selected hematological inflammatory markers accompany hepatic fat accumulation as part of the metabolic–inflammatory response. In this context, the TyG index appears to reflect the underlying metabolic disturbance driving steatosis severity rather than representing a secondary inflammatory marker.

From a clinical perspective, the TyG index represents a cost-effective adjunct to operator-dependent imaging modalities such as ultrasonography. In line with current clinical guidelines emphasizing early metabolic risk stratification [1], the TyG index may serve as a practical screening tool in outpatient settings. The stepwise increase in TyG values across steatosis grades also suggests potential utility for longitudinal monitoring, where reductions in TyG levels may reflect improvements in insulin sensitivity and hepatic fat burden. Furthermore, the high sensitivity observed at the identified cutoff value (7.90) supports the use of the TyG index as a rule-out tool to identify individuals who may benefit from further non-invasive evaluation.

Several limitations should be considered when interpreting the findings of this study. First, the retrospective cross-sectional design limits control over potential confounding factors and does not allow definitive causal inferences regarding the relationship between TyG index values and hepatic steatosis progression. Second, hepatic steatosis was assessed using ultrasonography rather than histology or advanced imaging techniques such as MRI–proton density fat fraction. Although examinations were performed by a single experienced radiologist to ensure internal consistency, ultrasonography may have reduced sensitivity for detecting mild fat infiltration. Third, while patients with overt diabetes mellitus and hypertension were excluded, individuals with prediabetes and dyslipidemia were included, resulting in a metabolically heterogeneous population.

As all participants had a body mass index ≥25 kg/m^2^, the presence of hepatic steatosis is consistent with the MASLD spectrum; however, the lack of prospectively collected anthropometric data, such as waist circumference or body composition measures, limited the evaluation of TyG-based composite indices (e.g., TyG-BMI or TyG–waist circumference). Additionally, detailed numerical body mass index values, waist circumference measurements, and contemporaneous blood pressure recordings were not consistently available in the electronic medical record system due to the retrospective design of the study. Therefore, these variables could not be included as baseline parameters in the statistical analysis. Furthermore, data on dietary habits and physical activity, which may influence triglyceride levels and insulin sensitivity, were unavailable due to the retrospective design. Moreover, fasting insulin measurements and insulin resistance indices such as HOMA-IR were not routinely available in the electronic medical record system and therefore could not be evaluated in this retrospective analysis.

Despite these limitations, this study has several important strengths. The inclusion of a relatively large, well-characterized cohort with standardized fasting laboratory measurements and uniform ultrasonographic assessment allowed a robust evaluation of the association between TyG index values and hepatic steatosis severity. However, the single-center design may limit the generalizability of the findings. Therefore, external validation in independent cohorts and multicenter studies is warranted to confirm the robustness and generalizability of the observed associations. Future prospective, multicenter studies incorporating detailed anthropometric data, lifestyle factors, and comparisons with established metabolic and steatosis indices are warranted to further validate these results.

## 5. Conclusions

In conclusion, this study demonstrates that the triglyceride–glucose (TyG) index increases progressively in parallel with the severity of hepatic steatosis within the metabolic dysfunction-associated steatotic liver disease (MASLD) spectrum. The observed graded relationship between TyG values and ultrasonographic steatosis severity, together with accompanying metabolic and inflammatory changes, supports the role of the TyG index as a practical surrogate reflecting the metabolic burden associated with hepatic fat accumulation. Given its wide availability, simplicity, and high sensitivity, the TyG index may serve as a useful tool for early risk stratification and screening in clinical practice, complementing imaging-based assessment in the evaluation of MASLD-related hepatic steatosis.

## Figures and Tables

**Table 1:** Baseline Characteristics of the Study Population.

Variable	*N* (Male: 147 Female: 333)	Minimum	Maximum	Mean	SD
**Age**	480	18	72	44.27	11.78
**Glucose (mg/dL)**	480	70	125	94.20	11.15
**Triglyceride (mg/dL)**	480	36	390	146.91	83.94
**TyG Index**	480	7.27	10.89	8.70	0.55
**HbA1c (%)**	480	4.80	6.50	5.74	2.33
**Platelets (×10^3^/µL)**	480	100	544	291.19	66.88
**Neutrophils (×10^3^/µL)**	480	1.91	9.85	4.63	1.42
**Lymphocytes (×10^3^/µL)**	480	0.42	5.87	2.50	0.76
**Monocytes (×10^3^/µL)**	480	0.18	1.08	0.48	0.15
**AST (U/L)**	480	8	125	23.84	12.18
**ALT (U/L)**	480	4	191	29.26	25.28
**Total Cholesterol (mg/dL)**	480	54	331	192.67	38.53

Data are presented as mean ± standard deviation (SD). Male: *n* = 147; Female: *n* = 333. TyG index: triglyceride–glucose index; AST: aspartate aminotransferase; ALT: alanine aminotransferase.

**Table 2:** Laboratory and Metabolic Parameters According to Steatosis Grades.

Variable	Grade 0 *n* = 160	Grade 1 *n* = 160	Grade 2–3 *n* = 160	*p* Value
**Glucose (mg/dL)**	90.7 ± 9.0	92 (71–122)	97 (76–125)	<0.001
**Triglyceride (mg/dL)**	86 (36–257)	135 (48–373)	189.5 (48–390)	<0.001
**TyG Index**	8.29 ± 0.42	8.74 ± 0.42	9.07 ± 0.49	<0.001
**HbA1c (%)**	5.40 (4.9–6.2)	5.60 (4.9–6.50)	5.76 ± 0.36	0.002
**Platelets (×10^3^/µL)**	276.5 (147–477)	282 (100–544)	288.5 (176–501)	0.038
**Neutrophils (×10^3^/µL)**	4.25 (1.91–7.40)	4.51 (2.12–8.64)	4.67 (2.56–9.85)	0.015
**Lymphocytes (×10^3^/µL)**	2.26 ± 0.65	2.36 (0.91–4.88)	2.81 ± 0.82	<0.001
**Monocytes (×10^3^/µL)**	0.435 (0.20–0.99)	0.450 (0.18–1.07)	0.480 (0.22–1.08)	0.004
**AST (U/L)**	20 (8–62)	21 (9–48)	22 (10–125)	<0.001
**ALT (U/L)**	17 (7–56)	21 (4–80)	28 (4–191)	<0.001
**Total Cholesterol (mg/dL)**	181.5 (54–331)	197.9 ± 38.8	196.0 ± 34.8	0.021

Data are presented as mean ± standard deviation or median (minimum–maximum), as appropriate. Grade 0: no steatosis; Grade 1: mild steatosis; Grade 2–3: moderate to severe steatosis. TyG index: triglyceride–glucose index; AST: aspartate aminotransferase; ALT: alanine aminotransferase. *p*-values were obtained using one-way ANOVA or Kruskal–Wallis tests, as appropriate.

**Table 3:** Comparison of Triglyceride–Glucose (TyG) Index Across Steatosis Grades.

Group	*n*	Mean ± SD	95% CI	Min–Max
**Grade 0**	160	8.29 ± 0.42	8.23–8.36	7.27–9.25
**Grade 1**	160	8.74 ± 0.42	8.68–8.81	7.67–9.75
**Grade 2–3**	160	9.07 ± 0.49	8.99–9.14	7.56–10.89
**Total**	480	8.70 ± 0.55	8.65–8.75	7.27–10.89

Data are presented as mean ± standard deviation (SD). TyG index: triglyceride–glucose index. Grade 0: no steatosis; Grade 1: mild steatosis; Grade 2–3: moderate to severe steatosis.

**Table 4:** Bonferroni Post Hoc Pairwise Comparisons.

Comparison	Mean Difference (I–J)	SE	*p*	95% CI
**Grade 0 vs. Grade 1**	−0.450	0.050	<0.001	−0.570 to −0.330
**Grade 0 vs. Grade 2–3**	−0.775	0.050	<0.001	−0.895 to −0.655
**Grade 1 vs. Grade 2–3**	−0.324	0.050	<0.001	−0.444 to −0.204

Mean difference represents the difference between the first and second group listed (I–J). *p*-values are Bonferroni-adjusted for multiple comparisons.

**Table 5:** Optimal Cutoff Value of the TyG Index for Hepatic Steatosis Based on ROC Analysis.

Parameter	Value
**Optimal cutoff (TyG)**	7.90
**Sensitivity**	0.984
**Specificity**	0.194
**Youden Index**	0.178

TyG index: triglyceride–glucose index. Cutoff value was determined using the maximum Youden index. Sensitivity and specificity correspond to this cutoff value derived from ROC analysis.

**Table 6:** Univariate and Multivariable Logistic Regression Analyses Identifying Factors Associated with Hepatic Steatosis.

Variable	B (Univariate)	OR (95% CI)	*p* Value	B (Multivariate)	Adjusted OR (95% CI)	*p* Value
**Age**	0.030	1.03 (1.01–1.05)	<0.001	0.013	1.01 (0.99–1.04)	0.294
**Male sex**	−0.756	0.47 (0.30–0.73)	0.001	−0.056	0.95 (0.52–1.72)	0.854
**Neutrophil count**	0.220	1.25 (1.08–1.44)	0.003	0.202	1.22 (0.99–1.52)	0.068
**Platelet count**	0.001	1.00(0.998–1.004)	0.357	-	-	-
**Lymphocyte count**	0.703	2.02 (1.52–2.69)	<0.001	0.245	1.28 (0.88–1.86)	0.200
**Monocyte count**	1.708	5.52 (1.43–21.36)	0.013	−0.399	0.67 (0.09–5.08)	0.699
**HbA1c**	2.027	7.59 (4.11–14.03)	<0.001	1.251	3.50 (1.57–7.77)	0.002
**AST**	0.049	1.05 (1.02–1.08)	<0.001	−0.024	0.98 (0.93–1.02)	0.298
**ALT**	0.045	1.05 (1.03–1.06)	<0.001	0.044	1.05 (1.02–1.07)	0.001
**TyG index**	2.956	19.22 (10.69–34.54)	<0.001	2.435	11.41 (6.10–21.34)	<0.001

**Abbreviations:** OR, odds ratio; CI, confidence interval; TyG index, triglyceride–glucose index; HbA1c, glycated hemoglobin; AST, aspartate aminotransferase; ALT, alanine aminotransferase.

## Data Availability

The data presented in this study are available on request from the corresponding author due to ethical and privacy restrictions.
